# Sound-Based Localization Using LSTM Networks for Visually Impaired Navigation

**DOI:** 10.3390/s23084033

**Published:** 2023-04-17

**Authors:** Mohsen Bakouri, Naif Alyami, Ahmad Alassaf, Mohamed Waly, Tariq Alqahtani, Ibrahim AlMohimeed, Abdulrahman Alqahtani, Md Samsuzzaman, Husham Farouk Ismail, Yousef Alharbi

**Affiliations:** 1 Department of Medical Equipment Technology, College of Applied Medical Science, Majmaah University, Al-Majmaah 11952, Saudi Arabia; 2 Department of Physics, College of Arts, Fezzan University, Traghen 71340, Libya; 3 Department of Biomedical Technology, College of Applied Medical Sciences in Al-Kharj, Prince Sattam Bin Abdulaziz University, Al-Kharj 11942, Saudi Arabia; 4 Department of Computer and Communication Engineering, Faculty of Computer Science and Engineering, Patuakhali Science and Technology, Patuakhali 6800, Bangladesh; 5 Department of Biomedical Equipment Technology, Inaya Medical College, Riyadh 13541, Saudi Arabia

**Keywords:** visually impaired people, sound source localization, indoor outdoor navigation, voice recognition, long short-term memory

## Abstract

In this work, we developed a prototype that adopted sound-based systems for localization of visually impaired individuals. The system was implemented based on a wireless ultrasound network, which helped the blind and visually impaired to navigate and maneuver autonomously. Ultrasonic-based systems use high-frequency sound waves to detect obstacles in the environment and provide location information to the user. Voice recognition and long short-term memory (LSTM) techniques were used to design the algorithms. The Dijkstra algorithm was also used to determine the shortest distance between two places. Assistive hardware tools, which included an ultrasonic sensor network, a global positioning system (GPS), and a digital compass, were utilized to implement this method. For indoor evaluation, three nodes were localized on the doors of different rooms inside the house, including the kitchen, bathroom, and bedroom. The coordinates (interactive latitude and longitude points) of four outdoor areas (mosque, laundry, supermarket, and home) were identified and stored in a microcomputer’s memory to evaluate the outdoor settings. The results showed that the root mean square error for indoor settings after 45 trials is about 0.192. In addition, the Dijkstra algorithm determined that the shortest distance between two places was within an accuracy of 97%.

## 1. Introduction

Numerous technologies are currently employed to enhance the mobility of the blind and visually impaired people (VIP). These technologies include the application of cameras, ultrasonic sensors, and computerized travel support. However, published statistics primarily classify visually impaired aids into two categories: indoor and outdoor. The indoor sensing technologies include laser, infrared, ultrasonic, and magnetic sensors. In comparison, outdoor sensing equipment includes the use of camera systems, intelligent navigation systems, GPS, mobile applications, carrying devices, robots, environment recognition systems, computer vision, and machine learning [1,2,3].

For indoor sensing devices, the distance between VIP and the surrounding objects is calculated by measuring the transmission and the receipt of some physical quality such as light, ultrasound, etc. [3]. However, this type of sensor enters little information about VIP. Outdoor sensing devices include a camera, smart navigation, GPS, mobile applications, carrying devices and robots, environment recognition systems, computer vision, and machine learning tools. These devices can enter more information than indoor methods. For example, the information could be about the environment surrounding the blind. Furthermore, these devices are more expensive than their indoor counterparts due to the processing phase, which needs computers or microprocessors [4,5].

Generally, blind individuals often face challenges when navigating their surroundings, both indoors and outdoors. Localization techniques for blind people are critical for their mobility, safety, and independence. The traditional localization techniques such as GPS or visual landmarks are not always accessible to blind people. To solve the problems of localization and interior navigation, several studies have been conducted [6,7,8]. For instance, Nagarajan et al. [9] developed a technique that used low-power Bluetooth-emitting devices. This technique was employed to locate various buildings according to their precise coordinates. This research work utilized an algorithm and different data formats as well as conducted experimental analyses to determine the optimal location for the beacons. Furthermore, this algorithm was implemented as an Android app that provided a navigational solution for the visually handicapped without relying on any external resources [9]. With the use of the Ultra-Wideband (UWB) location detection system, a spatial database of the environment for pathfinding via the application of the A* algorithm, and a guidance module, another study presented SUGAR, an internal navigation system for the visually handicapped. Through headphones, the user was communicated with via auditory signals and voice commands. A fully operational and user-friendly prototype was tested in the field with a visually impaired person to confirm the system’s viability for indoor navigation. Various other experiments were also carried out, all of which demonstrated the system’s accuracy [10]. 

Computer vision-based localization techniques are a system that uses cameras or other sensors to detect the user’s location and surroundings and provide real-time guidance based on that information. These systems can be effective in providing visual information to the user [11]. In one study, a smart framework called Vision Navigator was developed to help the visually impaired community by utilizing obstacle recognition, classification, and the real-time alerting of the user. The Smart-alert Walker and the Smart-fold Cane were the model’s building blocks. The Smart-fold Cane is a portable walking aid outfitted with sensors and cameras that identify and avoid any dangers in the user’s path. The recognition of obstacles is performed using the single-shot detection technique, and the recurrent neural network model converts the observed obstacle into text [12]. Another technique advocated for a smart setup, including a cyber-physical system with a human in the loop. More specifically, the system interpreted location context information through line-of-sight interaction based on visual signals and the distance sensing of material things. Information gleaned from social media platforms (Tweets) was also utilized to assess the general atmosphere of a given setting. The case study provided a more in-depth look at the proposed localization approaches (topological, landmark, metric, crowdsourced, and sound localization) and their applications in navigation, route verification, user tracking, socialization, and alerts [13]. 

Machine learning-based localization techniques are also a system that uses data from sensors and other sources to learn about the user’s behavior and preferences. The system can use this information to provide personalized recommendations and guidance to the user [6,14]. For instance, Ashiq et al. [15] developed to guide VIP based on a Convolution Neural Network (CNN) model. In this study, the method implemented a web-based application based on object detection and recognition. The user’s family was able to track the VIP via the sharing of the current location through this application. A different study was conducted by Tan et al. [16] to estimate the angle and distance using a sound-based localization technique. The method adopted CNN and regression model using the interaural phase difference (IPD). The system was tested with blind and visually impaired users and was determined to be effective in providing spatial information. Another study by Pang et al. [17] evaluated sound-based systems localization for visually impaired individuals using time-frequency with CNN. The system proposed to use multitask learning with extracted interaural time difference (ITD) and extracted interaural phase difference (IPD) from binaural signals. The experimental results of this method demonstrated that the localization performance is achieved under uncorrelated and diffuse noise conditions. 

The many advantages of these previous techniques have yet to solve the problem of real-time sensing. Their main weakness can be summarized as follows: traveling through uneven surfaces and unknown places is difficult for the blind. Traditional localization techniques, such as GPS or visual landmarks, are not always accessible to people who are blind or visually impaired. Therefore, researchers and developers have been exploring alternative techniques that leverage other sensory modalities, such as sound or vibration, to provide spatial information [18]. Modern technology, such as the integration of smartphone sensors, can identify complicated environments, discover new places, and direct users via voice commands to move to new places. This technology is inexpensive and reaches all blind, middle-income people [19,20]. However, the performance of these methods needs to be improved, especially when dealing with complex environments. Robots can fulfill the mission regarding capabilities and can cover all the required goals. Automated methods are very effective in complex navigation tasks, especially in new places as well as in global and local environments. Even though robots can provide useful information about obstacles, they are still limited in local and global markets and are still under clinical trials [21,22]. 

The issue of localization techniques for blind people highlights the need for continued research and development of innovative solutions that can provide accurate and reliable spatial information to individuals with visual impairments. Therefore, this work aimed to design and implement a sound-based localization technique that could guide and direct VIP to the right place in real time. In this technique, the system uses spatialized sound to provide information about the user’s location and surroundings. The user wears headphones or earbuds, and the system provides audio instructions based on their location. The design method considered the factors of safety and real-time processing in order to achieve independent movement, the identification of obstacles encountered by the blind in internal environments, and the ability to deal with new complex environments.

## 2. System Design

This work provides a simple, effective, and controllable electronic guidance system that helps VIP to move around in all predetermined places. The proposed method uses an integrated sonic system consisting of three ultrasonic sensors (indoor system). Its task is to gather information about obstacles in the blind lane by collecting all the reflective signals from the three sensors. Then, the software performs calculations in order to detect all the obstacles. In the case of external guidance (outdoor system), the proposed method is integrated with the positioning system to direct the visually impaired to predetermined places.

### 2.1. The System Architecture

**Figure 1:**
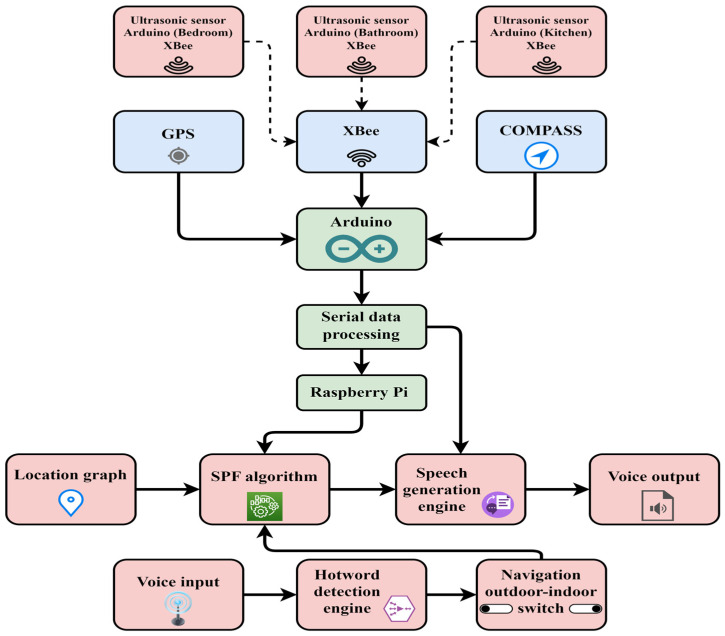
Illustrates the block diagram of proposed method.

Figure 1 illustrates the block diagram of the proposed method. In this work, the proposed method used a hybrid navigation system that included indoor/outdoor techniques [23]. The indoor system consisted of three ultrasound sensor networks: an Arduino Uno microcontroller, 3 XBEE Wi-Fi modules as end devices, and 1 XBEE as the coordinator. The three sensors were used in the kitchen, bathroom, and bedroom, with the possibility of increasing the number of sensors to any quantity. These sensors were used to capture the visually impaired person whenever they went through the ultrasonic range. Then, a high signal was sent via the XBEE end device module to the XBEE coordinator module, which was connected to the RaspberryPi4. As a result, the Raspberry Pi4 received a high signal with a known identifier number.

In this work, the visually impaired person in the outdoor system utilized voice commands with the use of hot keywords. The voice commands included system direction, and the coordinates of four places, including the supermarket, mosque, laundry, and house, were saved in the microcontroller memory for the outdoor system. In addition, the GPS coordinates (latitude and interactive longitude points) and the paths of the external map were also built. The outdoor devices consisted of a GPS, a digital compass, a speaker, and a microphone. The system had two modes (inside and outside); any mode could be activated by voice.

### 2.2. The Prototype Assembly 

**Figure 2:**
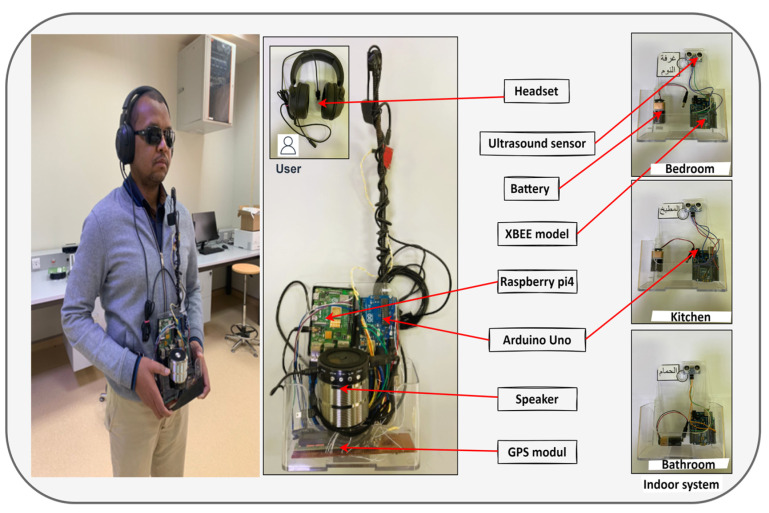
Prototype of the wearable smart system.

Figure 2 illustrates the prototype of the smart wearable system. The hardware of the system design consisted of the following:

- Ultrasound sensors (HC-SR04, Kuongshun Electronic, Shenzhen, China): Ultrasonic sensors are used to detect a person entering/leaving a room. The ultrasonic sensor is a piece of electronic equipment that uses the duration of the time interval between the sound wave traveling from the trigger and the wave coming back after colliding with a target.
- Arduino Uno microcontroller (ATmega328P, embedded chip, Microchip Technology Inc, Chandler, Arizona, USA): The microcontroller is used to collect ultrasonic, GPS, and compass data. Arduino is a microcontroller-based platform that uses an electronic environment and flash memory to save user programs and data. In the study, the Arduino module was used to read the input data that came from a different sensor.
- XBEE module s2 and wireless communication (771-6333, Digi International, Inc. Hopkins, MI, U.S): This is a radio frequency module (RF) that uses ZigBee mesh communication protocols (IEEE 802.15.4 PHY).
- Raspberry Pi4 (Single-board computer, Rockchip RK3399 CP, clocked up to 2 GHz, Creative Commons Corporation, Mountain View, CA, USA): As the central core of the system, this is used to receive data from different modules, such as Wi-Fi communications, via an XBEE module, or via a serial port such as a GPS, compass, microphone, etc.
- GPS module (HW-658 for Raspberry Pi, Dynamic—IT Solutions, Joppa, MD, USA): This is used to detect and locate user destinations. The GPS module (hw-658) is connected directly to the Arduino, which transfers GPS data to Raspberry Pi4 via a USB cable.
- Digital compass (HMC5883L chip, three-axis magnetic sensor, made by Honeywell Aerospace Inc., Phoenix, AZ, USA): This is a 3-axis digital compass that is used to determine the current direction of the module based on the magnetic field. The module must be kept in a horizontal position all the time. The study used the hmc5883l compass, which is suitable for Arduino applications.
- Headset (Razer Kraken Gaming Headset, Irvine, CA, USA): A headset is used for the audio communication between Raspberry Pi4 and the user so as to provide a good level of audio data transmission. The unidirectional microphone of the headset leaves no room for miscommunication and delivers crystal clear sound reproduction, with balanced, natural vocal tones and less background noise.

Table A1 in Appendix A illustrates all component specifications of hardware for the proposed system. 

## 3. Method

The system has two modes, and any of these modes could be activated vocally. The first mode is activated when the visually impaired person says the hot keyword “Inside”; then, the indoor navigation system tools start. The second mode is activated when the visually impaired says the hot keyword “Outside”; then, the system tools activate the GPS navigation tool, incorporating the external map previously saved in the microcontroller’s memory.

In this work, the blind person requests one set of location coordinates by saying the hot keyword through the microphone. After that, the system receives the audio file and then processes this file through the voice recognition software. It then converts the voice file into a text file. The application compares the text files with the previously saved location’s name. Supposing that the program detects a match in the values, it then starts collecting the location coordinates and subsequently sends this information to the blind person over the headset in the form of audio files, telling the blind how to reach the place. GPS is used to determine global positioning in real-time. The digital compass is used to determine the current direction in real time. During the movement of the blind person, the system helps to describe the direction to the desired location via a wireless headset (voice message). In addition, the blind person is alerted about the nearest objects.

### 3.1. The Indoor Navigation Algorithm

**Figure 3:**
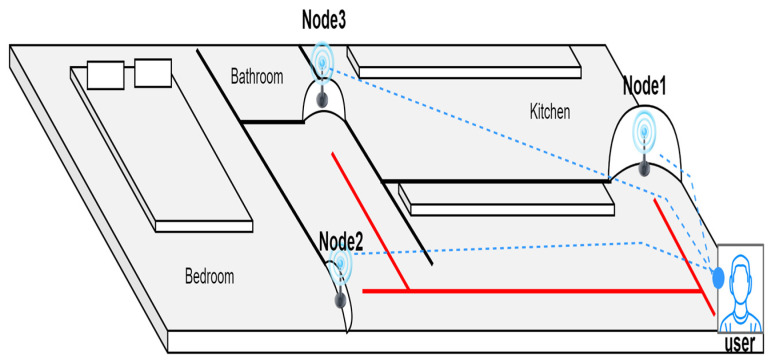
Location nodes of the indoor system.

The indoor system design consists of hardware hanging on the doors of three house rooms (kitchen “Node 1”, bedroom “Node 2”, and bathroom “Node 3”), as shown in Figure 3. Each part of the hardware is called a node XBEE module, and each module consists of an ultrasonic sensor, an Arduino module, an XBEE radio frequency module, and an XBEE shield. The ultrasonic ranging sensor is used to catch the visually impaired body if it is located within the sensor’s range. The Arduino Uno module reads the ultrasonic signal and calculates the distance between the blind and the sensor. If the distance between the visually impaired and the platform is less than 1.5 m, the microcontroller considers this as the visually impaired person approaching the detector. Then, the microcontroller sends a high signal via the XBEE module to the central Arduino held by the blind user; this Arduino is considered the coordinator microcontroller (coordinator XBEE module). The central Arduino then sends Raspberry Pi4 an identification code that was assigned to a specific router (the terminal XBEE modules), and Raspberry Pi4 recognizes the identification code that the programmer predefined. At this point, Raspberry Pi4 prepares an audio message (about the door in front of the blind), which is then sent to the blind person’s headset. This simple method allows for communication between the terminal units (kitchen, sleeping quarters, and bathroom) and the coordinator held by the blind user.

### 3.2. The Outdoor Navigation Algorithm

In outdoor locations, several predefined destinations were saved previously in the Raspberry Pi4 memory. The VIP requests for the coordinates of one location by saying the hot keyword through the microphone, which sends the system the audio file. Here, a voice recognition approach was adopted in order to produce a frequency map for each audio file. The long short-term memory (LSTM) model was also adopted to identify and filter out the output files.

### 3.3. Voice Recognition Approach 

In this approach, the Mel-frequency cepstral coefficients (MFCC) are used to extract the feature map of the audio file information [24,25]. Thus, in the extraction, a finite impulse response filter (FIR) is used for each audio file, as expressed by the following equation:

(1) γ(n)=r(n)−δr(n−1),

where γ(n) is the filter output, r(n) is the audio file, n is the number of samplings, and δ is given as (0<δ≤1).

To reduce signal discontinuity, framing and windowing (∅(n)) are employed as follows:

(2) $$\emptyset \left(n\right)=\{\begin{array}{cc} 1-\varepsilon \left(1+\cos\left(2\pi n/\Delta -1\right)\right) & n=0,1,\ldots ,\Delta -1\\ 0 & otherwise \end{array},$$

where ε and Δ are the constant and the number of frames, respectively. 

To determine each frame’s spectrum magnitude, fast Fourier transform (FFT) is applied as in the equation below:

(3) $$\gamma \left(k\right)=\sum_{n=0}^{\Delta -1}\gamma \left(n\right)e^{-j2\pi kn/\Delta },\;n=0,1,\ldots ,\Delta -1.$$

As a result, the Mel filter bank (f[m]) can be used as boundary points and be written as follows:

(4) $$f\left[m\right]=\left(N/F_{s}\right)B^{-1}\left(B\left(f_{l}\right)+m\frac{B\left(f_{h}\right)-B\left(f_{l}\right)}{M}\right),$$

where B(f)=1125ln((700+f)/700); $f_{l}$ is the lowest hertz and $f_{h}$ is the highest hertz; M and N are the number of the filter and the size of the FFT, respectively.

In this study, we employ an approximation homomorphic transform to eliminate the noise and spectral estimation errors, which is expressed as follows:

(5) $$\beta \left(m\right)=ln\left(\sum_{k=0}^{\Delta -1}\left|\gamma \left(k\right)f\left[m\right]\right|\right).$$

In the final step of the MFCC processing, we recall the discrete cosine transformer (DCT) function in order to obtain high decorrelation properties for the system, which is carried out as follows:

(6) $$d_{l}\left(n\right)=\sqrt{2/M}\sum_{m=1}^{M}\beta \left(m\right)cos\left(\frac{n\pi }{M}\left(m-0.5\right)\right),\;n=0,1,2,\;\ldots ,\;l<M.$$

The system feature map is achieved by taking the first and second derivatives of Equation (6). As a result, the LSTM creates and utilizes the database, which is applicable to all recordings that were made.

### 3.4. LSTM Model Adoption 

**Figure 4:**
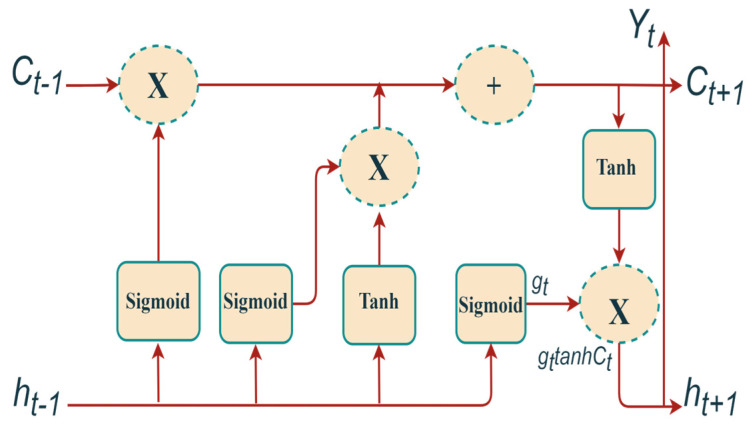
Structure of LSTM model.

A vanilla LSTM structure is adopted to classify the spectrum file [26,27,28]. The model architecture is composed of several memory block-style sub-networks that are continuously connected to each other as shown in Figure 4. The model consists of a cell, an input gate, an output gate, and a forget gate. In this model, a sigmoid function (σ) is used to identify and eliminate the current input ($q_{t}$) and the last output ($y_{t-1}$) data. This can be achieved by using the forgetting function gate ($g_{t}$), as expressed by the equation below:

(7) $$g_{t}=\sigma \left(w_{f}\left(y_{t-1},q_{t}\right)+j_{f}\right),$$

where $w_{f}$ represents the weight matrices, and $j_{f}$ is the bias weight vector.

By using the sigmoid layer and the tanh layer, the model is required to store the new input data and then update that data in the cell state ($C_{t}$) as follows:

(8) $$C_{t}=C_{t-1}g_{t}+H_{t}R_{t},$$

where $H_{t}=\sigma \left(w_{i}\left(y_{t-1},q_{t}\right)+j_{i}\right)$, and $R_{t}=tanh\left(\left(w_{c}\left(y_{t-1},q_{t}\right)+j_{c}\right)\right)$. 

As a result, the output value is provided as follows:

(9) $$y_{t}=g_{t}\tan h\left(C_{t}\right).$$

### 3.5. Dijkstra SPF Algorithm 

**Figure 5:**
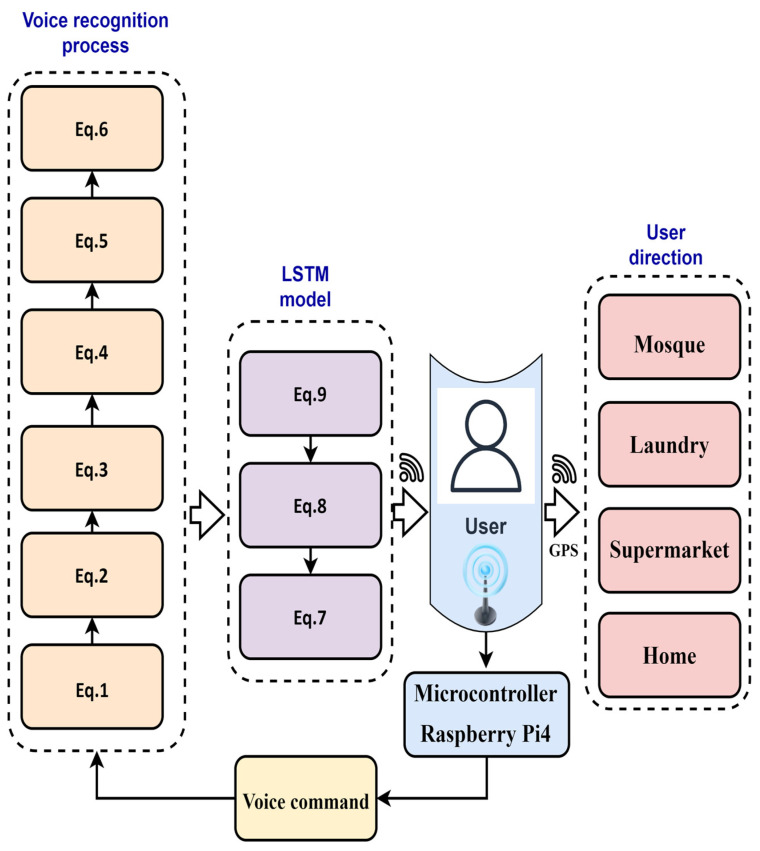
Flowchart of the system.

This algorithm is used to calculate the shortest distance between two points (shortest path first, SPF) [29,30]. The coordinate path is saved in matrix form. As a result, whenever the user activates any path, the Dijkstra SPF algorithm calls the priority queue tool. This tool compares the elements and selects the one with high priority before the element with low priority. The below-described Algorithm 1 was implemented to accomplish this process. Figure 5 illustrates the flowchart of outdoor system.
**Algorithm1:** Dijkstra SPF**Input**: “voice command data”**Output**: GPS “SPF”1: Start2: Initialization: *{distances to source node (s) = 0; distances to other nodes is empty (n) = ∅; queue (q) ∈ {all nodes}}*3: *Start value ← 0*4: **for** *all N ∈ n-{s}*5: *dist [n] ← ∞ (all other distances should be set to infinity)*6: *while q ≠ ∅ (while there is a queue)*7: **do** *x ← min_distance (q, dist) (choose the q with the lowest distance)*8: *For all N ∈ neighbors [x]*9: **do** *if dist [N] > dist [x] + w (x, N) (if a new shortest path is discovered)*10: **then**  *d[v]← d[x] + w (x, N) (change the shortest path with new value)***Return** *dist*

## 4. Simulation Protocols and Evaluation Methods

Python and the C++ software were used to control the algorithms in the hardware [31]. An English speech group consisting of separate words, provided by the Health and Basic Sciences Research Center at Majmaah University, was used to evaluate the proposed method. The correct pronunciation of all 3500 words included in the group was derived for 7 fluent Arabic speakers. Data were recorded at a sampling rate of 25 kHz, with a resolution of 16 bits. Speed, dynamic range, noise as well as forward and backward time shifts were subsequently adjusted. Approximately 80% of the samples (2800) were used to create the training set (training and validation), while the remaining 20% was used to create the test set (700). All trials were carried out for a total of 50 epochs, and there were 4 participants in each batch.

In order to verify the accuracy of target detection within the ultrasound range for the indoor experiment, the root mean square error (RMSE) was used to compare observed ($X_{o}$) and predicted ($X_{p}$) values:

(10) $$RMSE=\sqrt{\left(\overset{n}{\underset{i=1}{\sum }}{\left[X_{o,i}-X_{p,i}\right]}^{2}/n\right).}$$

As for the outdoor experiment, in order to provide a measure of the quality and accuracy of the proposed system’s predictions, we computed the F-score with precision (p) and recall (r) using the following formula:

(11) F=2[p∗r/(p+r)],

where $p=\left[t_{p}/\left(t_{p}+f_{p}\right)\right]$ and $r=\left[t_{p}/\left(t_{p}+f_{n}\right)\right]$; here, $t_{p}$, $f_{p}$, and $f_{n}$ are the true positive, false positive, and false negative, respectively. 

The coordinate paths and nodes of four places outside the house were saved in the microcontroller memory. Each path contains a different number of nodes (latitude, longitude), and the number of these interactive points is based on the distance between the start node and the destination node.

## 5. Results

### 5.1. Indoor System Test

This mode was activated through indoor navigation tools whenever the visually impaired person said the hot keyword “inside”. Three ultrasonic sensors were placed in the kitchen, bedroom, and bathroom to ensure the indoor system worked perfectly. To perform this experiment, we chose three participants who were 18–50 years in age and 90–150 cm in height. Each participant repeated the experiment fifteen times. During the experiment, the participants were sent a voice message through the headset, telling them the specific room toward which they were headed, as shown in Table 1. Then, the information from the internal ultrasonic sensors was sent to the Raspberry Pi4; each ultrasonic device had its own IP address, XBEE: ID.

For example, suppose the participant was headed in the direction of the kitchen. In that case, the ultrasound sensor near the kitchen door would pick up the movement of the object and send this information to the Raspberry Pi4 located in the tools used by the participant. Then, the Raspberry Pi4 would generate voice messages that tell the blind person where they were at that moment.

Table 2 depicts the accuracy of target detection within the ultrasound range. Based on another experiment’s results, the accuracy ratio is high enough. The root mean square error for the three cases is equal to 0.192.

### 5.2. Outdoor System Test

**Figure 6:**
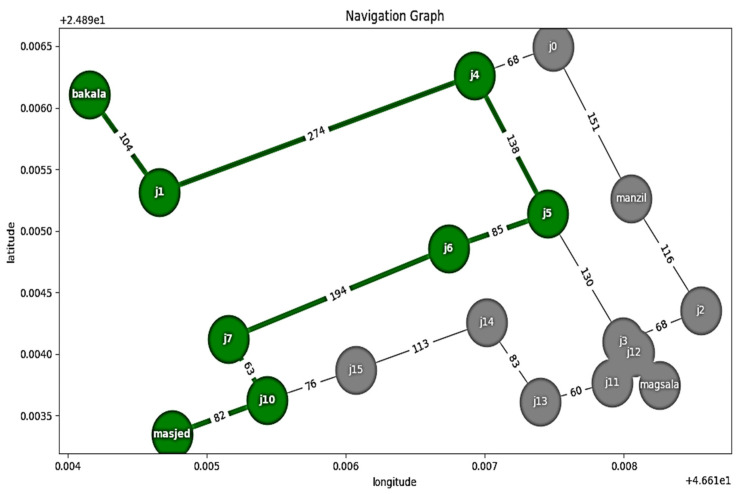
Outdoor mode navigation.

In this test, the outdoor mode was activated through navigation tools whenever the visually impaired said the hot keyword “outside”. As a result, three paths were saved in the Raspberry Pi4 memory (“from home to a mosque”; “from home to laundry”; “from home to Supermarket”). In addition, the latitudinal and longitudinal nodes located at different distances along the path were also saved, as shown in Figure 6. 

The processing started with the conversion of the audio file waves into their frequency domain using Fourier analysis. Then, these frequency domain waves were converted into spectrograms and used as input for the LSTM model. The confusion matrix was constructed with the help of the preliminary findings, as can be seen in Table 3. When considering all four voice commands, the average accuracy was approximately 97% of the accurate forecast. To provide a clearer picture of the classification process, we used the terms “true positives”, “true negatives”, “false positives”, and “false negatives”. Table 4 displays the results of the computations for the ratio of the voice-command predictions, as well as those for accuracy and precision.

**Figure 7:**
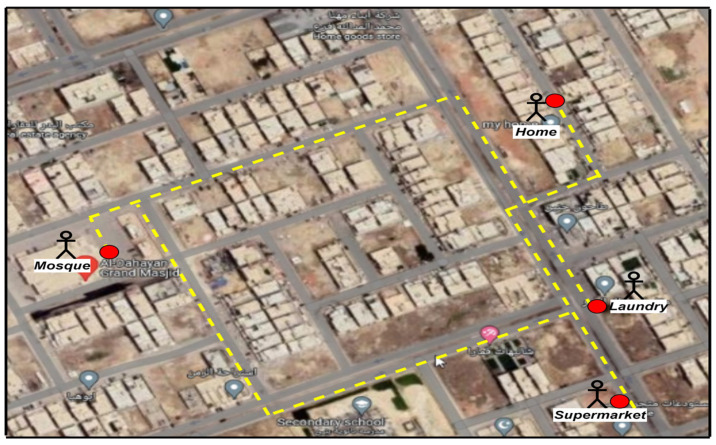
Navigation using the outdoor map (home to laundry).

Figure 7 shows the mode of the outdoor map where the evaluation occurred (Al-Arid district, Riyadh City). In this mode, the visually impaired attempted to use the path from home to the laundry, as shown in Figure 7, with the coordinates given in Table 5. The system provided a map with 49 nodes, which extended from the start node (home) to the end node (laundry). The distance between each node was between 1 and 3 m. Instructions on how to keep moving down the path were sent to the visually impaired person. The system presented the location and provided advice, and family members who joined the journey followed the blind person all the way. The visually impaired person walked and received the GPS data and the digital compass navigation via a headset. All the nodes through which the visually impaired had passed were recorded.

### 5.3. Outdoor Shortest Path First (SPF)

**Figure 8:**
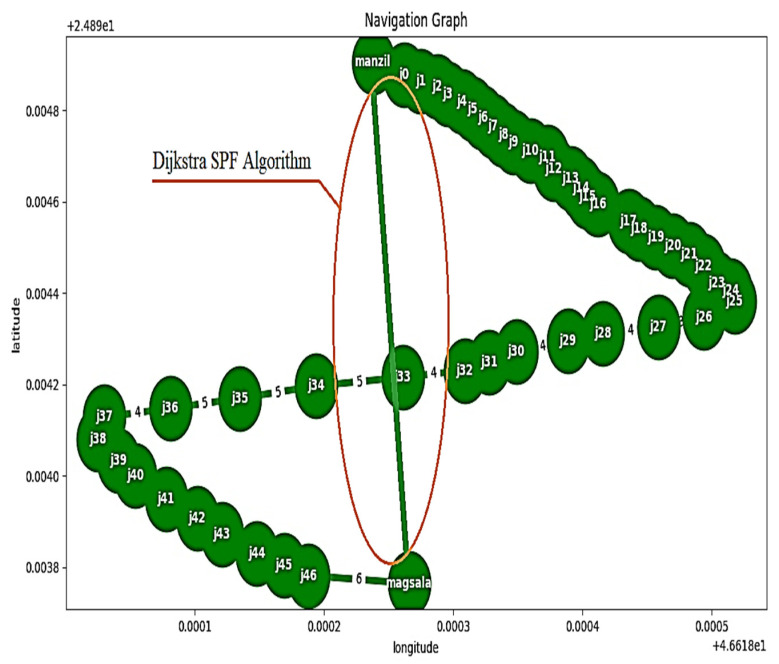
The shortest path first (SPF) algorithm used to determine the shortest path between points.

The Dijkstra SPF algorithm was set to work in the automatic mode. As a result, the shorter distance between any of the places could be determined through the predefined nodes. By applying the Dijkstra algorithm, the calculation for the shortest path between two nodes (home and laundry) could be performed, as shown in Figure 8. The algorithm was tested in three trials. Table 6 illustrates that the Dijkstra algorithm can easily discover the shortest distance between two places.

## 6. Discussion

Sound-based systems provide a means of localization for visually impaired individuals that rely on auditory cues. These systems use sound to provide users with information about their environment and their location in space. Sound-based systems have the advantage of being relatively low-cost and easy to implement. However, the effectiveness of these systems depends on the user’s ability to interpret auditory cues and the quality of the sound-based system used [23]. 

This study aimed to develop and implement a robust and affordable sound localization system for aiding and directing people with visual impairments. This concept was conceived to help blind people become significantly more independent, ultimately improving their quality of life. The suggested innovative wearable prototype expands the capabilities of existing system designs by integrating cutting-edge intelligent control systems such as speech recognition, LSTM model, and GPS navigation technologies. Voice recognition technology, wireless network systems, and considerable advances in sensor technologies have all contributed to the widespread adoption of navigation technology for guiding blind individuals.

The proposed system is generally characterized by the simplicity with which the electrical and electronic circuits can be installed, as well as by its low cost and low energy consumption. The prototype of a straightforward electronic circuit connection is shown in Figure 2. Regarding the materials and methods utilized, their ability to be modified, personalized, and then conveyed to the end-user, the design is both highly efficient and inexpensive. It is possible to avoid every impediment with an average response time of about 0.5 s when processing a single task. This smart prototype’s programs and applications can all function without an internet connection. Additionally, the suggested software operates with great precision even when there is outside noise.

For indoor navigation, the study investigated the accuracy of detecting the desired object. By using voice commands, the user could navigate to the right destination; the RMSD was used to represent the navigational errors. The experimental results exhibited a significantly high root mean square error ratio. As can be seen in Table 2, the average RMSE after 45 experimental trials using ultrasonic sensor detection is 0.192. The system also exhibited a high prediction ratio via a normalized confusion matrix, as presented in Table 3. In addition, the results presented in Table 4 for the accuracy, precision, recall, and F-score of the voice commands demonstrate that the designed system works efficiently. The Dijkstra algorithm was also developed and incorporated into the designed system in order to determine the shortest distance between any two places. By using the Dijkstra algorithm, the system was able to detect the shortest distance with an accuracy of 99%.

Based on comparisons to prior studies on efficacy, reliability, and cost, we believe that our design and implementation approach in this study has addressed numerous complexities. For example, a recent study conducted by Ramadhan, A.J. [32] implemented and tested a wearable smart system to help VIP based on sensors that track the path and alert the user of obstacles. To carry out the performance with high accuracy, the design had to be able to produce a sound emitted through a buzzer and vibrations on the wrist. Furthermore, this system depended on other people and sent SMS messages to family members for additional assistance. A different study used the Uasisi system to assist VIP. In this system, the modular and adaptable wearable technique was implemented. The authors incorporated the vibratory feedback mode with cloud infrastructure to sense the proximity of objects and navigate the patterns of the user. Although this work was evaluated and tested, it is still in the initial stages and needs to add more sensors to detect obstacles in the user’s environment [33].

In general, the system performance of sound-based localization for visually impaired individuals can be affected by the presence of multiple speakers in the surrounding environment. Thus, the ability of the system to accurately localize the target sound source can be increased in the presence of competing sounds or background noise. To achieve this goal, the present study focused on developing voice recognition algorithms techniques using MFCC and LSTM to improve the robustness of the proposed system. The study results demonstrated that the system achieved accuracy of 97% when the signal-to-noise ratio (SNR) was at a minimum of 15.5 dB (refer to Table A1 in Appendix A). On the hand, by comparing localization techniques, a quantitative metric such as accuracy is often used to measure the performance of each method. This metric can be used to evaluate each technique’s effectiveness and determine which performs best for a specific application. Table 7 summarizes the technique, method, and accuracy reported in different studies using different approaches. Compared with our study, implementing ultrasonic-based systems with the LSTM model is a promising solution for the localization of visually impaired individuals. These systems showed promise in providing location information and detecting obstacles in real-world environments.

In addition, this study presented a power consumption of a system designed for indoor and outdoor localization to estimate its lifetime when integrated into an assistive device. The system is evaluated regarding power consumption, with measurements taken for indoor and outdoor environments. Results indicate that the power consumption of outdoor localization is higher than that of indoor localization, with an average of 1.5 and 1 watt, respectively. The system’s lifetime is then estimated based on the battery capacity of the assistive device, with the analysis revealing that the system can run for approximately 5 h in outdoor environments and 7 h indoors. These findings provide important insights for the development of assistive devices that incorporate localization systems, ensuring that they can operate effectively for extended periods in various environments.

## 7. Conclusions

In this work, sound-based localization prototype was developed to automatically guide the blind and VIP. Software and hardware tools were used to implement the proposed prototype. Assistive hardware tools, including Raspberry Pi4, ultrasound sensors, the Arduino Uno microcontroller, an XBEE module, a GPS module, a digital compass, and a headset, were utilized to implement this method. Python and the C++ software were developed through the use of robust algorithms in order to control the hardware components via an offline Wi-Fi hotspot. To train and identify various voice commands such as mosque, laundry, supermarket, and home, a built-in voice recognition model was created using the LSTM model. The Dijkstra algorithm was also adopted to determine the shortest distance between any two places. 

The simulation protocols and evaluation techniques used three thousand five hundred varied word utterances recorded from seven proficient Arabic speakers. Data recording was performed at a resolution of 16 bits and a sampling rate of 25 kHz. The accuracy, precision, recall, and F-score for all the voice commands were computed with a normalized confusion matrix. The results from the actual testing showed that controlled interior and outdoor navigation algorithms have a high degree of accuracy. Furthermore, it was shown that the calculated RMSD between the intended and actual nodes during indoor/outdoor movement was accurate. To conclude, the realized prototype is simple, inexpensive, independent, secure, and also includes other benefits. 

## Figures and Tables

**Table 1:** Indoor destination information (feedback for the system).

Raspberry Voice Command	IP Address Detected
You are going to the bedroom	Bedroom
You are going to the kitchen	Kitchen
You are going to the bathroom	Bathroom

**Table 2:** Detection ratio of the ultrasonic sensor (human participants).

Number of Trials	Rooms Name					
	Kitchen		Bedroom		Path Room	
	Detected	Undetected	Detected	Undetected	Detected	Undetected
1	1	0	1	0	1	0
2	1	0	1	0	1	0
3	1	0	1	0	1	0
4	1	0	1	0	1	0
5	0	1	1	0	1	0
6	1	0	1	0	1	0
7	1	0	1	0	1	0
8	1	0	1	0	1	0
9	1	0	0	1	1	0
10	1	0	1	0	1	0
11	1	0	1	0	1	0
12	1	0	1	0	1	0
13	1	0	1	0	1	0
14	1	0	1	0	1	0
15	1	0	1	0	1	0
Root means square error	0.192					

**Table 3:** Normalized confusion matrix.

Actual Voice Command					
Prediction ratio %	Class	Mosque	Laundry	Supermarket	Home
	Mosque	96%	1%	2%	3%
	Laundry	1%	98%	2%	2%
	Supermarket	2%	2%	97%	1%
	Home	3%	2%	1%	96%

**Table 4:** Accuracy, precision, recall, and F-score for voice commands.

Class	Accuracy	Precision	Recall	F-Score
Mosque	95%	0.73	0.74	0.735
Laundry	96.3%	0.75	0.75	0.75
Supermarket	98.2%	0.77	0.75	0.76
Home	94.8%	0.75	0.73	0.74

**Table 5:** Example for one student participant and one destination.

Planned Longitude	Planned Latitude	Actual Longitude	Actual Latitude
24.89482	46.61831	24.89499	46.618357
24.894803	46.61832	24.89485	46.618365
24.894784	46.61832	24.894799	46.618373
24.894765	46.61833	24.894795	46.618381
24.894748	46.61834	24.894792	46.618389
24.894731	46.61835	24.894787	46.618387
24.894712	46.61836	24.894712	46.61836
24.894697	46.61837	24.894697	46.618393
24.894673	46.61838	24.894699	46.618398
24.894651	46.61839	24.894689	46.618391
24.894629	46.6184	24.894679	46.618399
24.894612	46.6184	24.894662	46.618454
24.894595	46.61841	24.894595	46.618442
24.894558	46.61844	24.894598	46.618466
24.894541	46.61844	24.894589	46.618474
24.894522	46.61846	24.894572	46.618477
24.894503	46.61847	24.894503	46.61847
24.894484	46.61848	24.894494	46.618493
24.89446	46.61849	24.89486	46.618494
24.894423	46.6185	24.894463	46.618544
24.894406	46.61852	24.894456	46.618555
24.894382	46.61852	24.894392	46.618558
24.894348	46.61849	24.894388	46.618494
24.894326	46.61846	24.894366	46.618499
24.894311	46.61842	24.894361	46.618446
24.894296	46.61839	24.894296	46.618399
24.894272	46.61835	24.894262	46.618389
24.89425	46.61833	24.89475	46.618358
24.894231	46.61831	24.894271	46.618339
24.894216	46.61826	24.894266	46.618291
24.894197	46.61819	24.894157	46.618194
24.89417	46.61814	24.89417	46.618135
24.894148	46.61808	24.894178	46.618081
24.894129	46.61803	24.894129	46.61803
24.89408	46.61803	24.89408	46.618025
24.894034	46.61804	24.894094	46.618061
24.894	46.61805	24.894	46.618084
24.893949	46.61808	24.893989	46.618098
24.893908	46.6181	24.893948	46.618142
24.893872	46.61812	24.893892	46.618151
24.893831	46.61815	24.893891	46.618178
24.893804	46.61817	24.893854	46.618189
24.893782	46.61819	24.893792	46.618198
24.893765	46.61827	24.893785	46.618296

**Table 6:** Dijkstra shortest path first (SPF) algorithm.

Expected Tracks	Found the Shortest Distance (Dijkstra)	Could Not Find the Shortest Distance (Dijkstra)
Home to mosque	738 m	----
Home to supermarket	561 m	----
Home to laundry	214 m	----
Mosque to home	738 m	----
Laundry to home	214 m	----
Supermarket to home	561 m	----
Mosque to supermarket	940 m	----
Mosque to laundry	584 m	----
Laundry to supermarket	676 m	----
Laundry to mosque	584 m	----
Supermarket to mosque	940 m	----

**Table 7:** Summarizing the technique, method, and accuracy for different studies.

Authors	Technique	Method	Average Accuracy
Tan et al. [16]	Interaural phase difference (IPD)	Convolutional Neural Network and Regression Model	98.96%–98.31%
Pang et al. [17]	ITD, IPD, and Microphone-array geometry	Time–frequency convolutional neural network (TF-CNN)	90%
Yiwere et al. [34]	Labeling of audio data	Deep Learning: An Image Classification	88.23%
Hu et al. [35]	Relative harmonic coefficients	Semi-supervised multi-source algorithm	50% to 90%
MA et al. [36]	Phased microphone array	Deep Learning	70.8%–100%
Rahman et al. [37]	Ultrasonic and PIR motion sensor	Obstacle and Fall Detection based on Bluetooth	98.34%
Sipos et al. [38]	RFID reader	Obstacle detection	40%–99%
AL-Madani et al. [39]	Bluetooth Low Energy (BLE) beacons	Fuzzy Logic Type-2	98.2%

## Data Availability

Not applicable.

## Future Work

Further research is needed to evaluate the effectiveness of these systems in different environments and scenarios.

## Appendix A

The following tables show the component specifications of hardware for the proposed system (Table A1) and the ultrasound sensor (HC-SR04) specification with our study results (Table A2).

**Table A1:** The hardware specifications.

Parameter	Value
Raspberry pi4	SoC: quad-core, 64-bit @ 1.5 GHz. Networking: 2.4 GHz and 5 GHz wireless LAN. Bluetooth: Bluetooth 5.0 RAM: 4 GB SDRAM. GPU: Broadcom Video Core VI. Storage: microSD.
Ultrasound sensors	Supply Voltage: 5 V. Current Consumption: 15 mA. Frequency: 40 kHz. Trigger Pulse Width: 10 µs.
Arduino Uno mi-controller	ATmega328P: (embedded chip). Analog inputs: 6. Digital input/output pins: I14. Connection: USB.
XBEE module s2	1 MB / 128 KB RAM (32 KB are available for Micro Python)
GPS module	Speed precision: <0.1 m/s. Capturing sensitivity: −148 dm. Voltage: 3.3–5 V. Location precision: <2.5 m CEP.
Digital compass	Voltage: 3–5 V Measuring range: ±1.3–8 gauss.
Headset	Min Frequency Response: 20 Hz. Max Frequency Response: 20 kHz. Impedance: 32 Ohm. Power Input: 50 mW. Sound Features: Sensitivity 112 dB/mW.

**Table A2:** The proposed indoor/ outdoor accuracy evaluation.

Sensor Specification	Sensor type	Ultrasound sensor: HC-SR04	
	Target Object	Human	
	Operating min range	2 cm	
	Operating max range	400 cm	
	Operating frequency range	40 kHz	
Study result	The required targeting range	The dead zone is <0.5 cm > 151 cm. The active zone is <150 cm >1 cm	
	Participant numbers	35 in active zone	35 in dead zone
	Positive	34	1
	Negative	1	34
	Accuracy	97%	
